# Expression and Function of Cannabinoid Receptors CB1 and CB2 and Their Cognate Cannabinoid Ligands in Murine Embryonic Stem Cells

**DOI:** 10.1371/journal.pone.0000641

**Published:** 2007-07-25

**Authors:** Shuxian Jiang, Yigong Fu, John Williams, JodiAnne Wood, Lakshmipathi Pandarinathan, Shiri Avraham, Alexandros Makriyannis, Shalom Avraham, Hava Karsenty Avraham

**Affiliations:** 1 Division of Experimental Medicine, Beth Israel Deaconess Medical Center, Harvard Medical School, Boston, Massachusetts, United States of America; 2 Center for Drug Discovery, Northeastern University, Boston, Massachusetts, United States of America; Baylor College of Medicine, United States of America

## Abstract

**Background:**

Characterization of intrinsic and extrinsic factors regulating the self-renewal/division and differentiation of stem cells is crucial in determining embryonic stem (ES) cell fate. ES cells differentiate into multiple hematopoietic lineages during embryoid body (EB) formation *in vitro*, which provides an experimental platform to define the molecular mechanisms controlling germ layer fate determination and tissue formation.

**Methods and Findings:**

The cannabinoid receptor type 1 (CB1) and cannabinoid receptor type 2 (CB2) are members of the G-protein coupled receptor (GPCR) family, that are activated by endogenous ligands, the endocannabinoids. CB1 receptor expression is abundant in brain while CB2 receptors are mostly expressed in hematopoietic cells. However, the expression and the precise roles of CB1 and CB2 and their cognate ligands in ES cells are not known. We observed significant induction of CB1 and CB2 cannabinoid receptors during the hematopoietic differentiation of murine ES (mES)-derived embryoid bodies. Furthermore, mES cells as well as ES-derived embryoid bodies at days 7 and 14, expressed endocannabinoids, the ligands for both CB1 and CB2. The CB1 and CB2 antagonists (AM251 and AM630, respectively) induced mES cell death, strongly suggesting that endocannabinoids are involved in the survival of mES cells. Treatment of mES cells with the exogenous cannabinoid ligand Δ^9^-THC resulted in the increased hematopoietic differentiation of mES cells, while addition of AM251 or AM630 blocked embryoid body formation derived from the mES cells. In addition, cannabinoid agonists induced the chemotaxis of ES-derived embryoid bodies, which was specifically inhibited by the CB1 and CB2 antagonists.

**Conclusions:**

This work has not been addressed previously and yields new information on the function of cannabinoid receptors, CB1 and CB2, as components of a novel pathway regulating murine ES cell differentiation. This study provides insights into cannabinoid system involvement in ES cell survival and hematopoietic differentiation.

## Introduction

Murine embryonic stem (mES) cells, derived from the inner cell mass of preimplanted embryos, are pluripotent and retain the ability to differentiate into cells of all three germ layers of the developing mouse embryo. Understanding the regulatory mechanisms responsible for the hematopoietic differentiation of mES cells is crucial in defining the pathways and molecular events that control germ layer determination and tissue formation.

ES cells also exhibit the capacity to contribute to a wide range of well-defined cell types when using several *in vitro* models of differentiation. *In vitro* differentiation assays using ES cultures involve the removal of Leukemia inhibitory factor (LIF) and separation of the cells from the feeder layer under conditions that promote the formation of embryonic stem cell aggregates, termed embryoid bodies (EBs). These EBs contain a number of different cell types [1]–[2]. Molecular assays in combination with *in vitro* differentiation assays of ES cells provide insights into the early molecular events associated with lineage specification.

Although the *in vitro* hematopoietic differentiation of ES cells has been characterized at both the cellular and molecular levels, the pathways that regulate the hematopoietic differentiation of ES cells are not well defined [3], [4]. ES cells can be expanded *ex vivo* as undifferentiated cells that retain a normal karyotype or, alternatively, can be differentiated *ex vivo* into cell types of all three germ layers [2]. LIF is required to maintain the undifferentiated state of ES cells, whereas withdrawal of LIF initiates the formation of EBs and cellular differentiation [3], [4]. Even though EBs are far less organized than the actual embryo, they can partially mimic the spatial organization in the embryo. The developmental mechanisms of vascular and hematopoietic systems in EBs are similar to those in the yolk sac [5]–[8].

G-coupled protein receptor (GPCR) members play a central role in regulating the spatial distribution of immature and mature hematopoietic cells, including their release into the circulation and homing to hematopoietic tissue. GPCRs have been linked to many functions, including cell proliferation, maturation, survival, apoptosis, and migration [9]–[12]. The CB1 and CB2 cannabinoid receptors are members of the GPCR family. The CB2 receptors are primarily expressed in myeloid, macrophage, erythroid, lymphoid and mast cells [13]. The brain cannabinoid receptor CB1 is also expressed in hematopoietic cells such as lymphocytes, splenocytes and T cells, but mostly CB1 receptors are expressed at high levels in the central nervous system (CNS) where they regulate the attenuation of synaptic transmission and psychoactivity [14]–[20]. To date, several endogenous lipids that are derivatives of long-chain fatty acids have been isolated and characterized as natural ligands, and are termed endocannabinoids. Endocannabinoids are synthesized *in vivo* by various tissues on demand through cleavage of membrane precursors, and are involved in short range signaling processes [21]. Four types of endogenous compounds have been discovered so far and been proposed to act as endocannabinoids: 1) anandamide (AEA) (*N*-arachidonoyl-ethanolamine) and some of its derivatives; 2) 2-arachidonoylglycerol (2-AG) and noladin ether (2-arachidonoyl glycerol ether); 3) virodhamine (*o*-arachidonoyl-ethanolamine); and 4) *N*-arachidonoyl-dopamine (NADA). Since their discovery, endocannabinoids, anandamide and 2-AG in particular, have been implicated in physiological functions as well as in many pathological conditions. Endocannabinoids have been isolated from the brain as well as from the spleen and other peripheral tissues [21]. The presence of endocannabinoids in hematopoietic and immune cells suggests that CB2 and its endogenous ligands may play critical physiological roles in the regulation of inflammatory reactions and immune responses [22]. However, the expression, function and the precise roles of CB1 and CB2, as well as their cognate ligands, in ES cells are unknown.

Natural cannabinoids are the constituents of marijuana plants [23]. Δ^9^-tetrahydrocannabinol ( = THC), a major psychoactive constituent of marijuana, interacts with both the CB1 and CB2 receptors, thereby eliciting a variety of pharmacological responses *in vitro* and *in vivo* [24]. Many agonists have been developed that are selective for the CB1 (ACPA, ACEA) and CB2 (JWH-015, JWH-133) receptors and have significantly higher affinities for one receptor over the other [24]–[29]. Furthermore, various antagonists that specifically inhibit the CB1 or CB2 receptors have also been developed. Anandamide and 2-AG are endogenous ligands, members of the eicosanoid class of cannabinoids, which are arachidonic acid derivatives and are structurally different from other cannabinoid classes.

We hypothesize that CB1 and CB2 play regulatory roles in the hematopoietic differentiation of ES cells and that endocannabinoids are important for the survival of ES cells. Here, we examined the expression and function of CB1 and CB2 in mES cells and determined their role in mES cell hematopoietic differentiation. We also analyzed the expression of endocannabinoids in mES cells and determined the effects of cannabinoid antagonists on ES cell survival.

## Results

### Expression of CB1 and CB2 in murine embryonic stem cells and murine embryoid bodies

**Figure 1:**
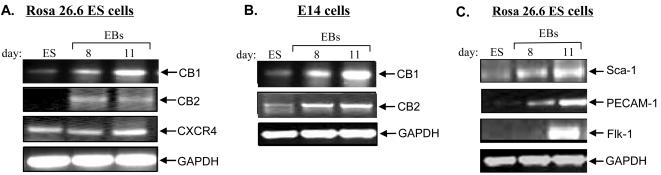
Expression of CB1 and CB2 in Rosa26.6 (Panel A) and E14 (Panel B) ES cells. Cells were washed with PBS, and then RNA was isolated and analyzed by RT-PCR using specific primers for CB1, CB2, GAPDH and CXCR4. Panel C: RT-PCR analysis of the *in vitro* differentiation of Rosa26.6 ES cells, using specific primers for GAPDH, Flk-1, PECAM-1 and Sca-1. EBs: Embryoid bodies. ES cells: undifferentiated control ES cells. The following primers were used: GAPDH: 292 bp S 5′-CTCACTGGCATGGCCTTCCG-3′ AS 5′-ACCACCCTGTTGCTGTAGCC-3′ CB1: 430 bp S 5′-CGTGGGCAGCCTGTTCCTCA-3′ AS 5′-CATGCGGGCTTGGTCTGG-3′ CB2: 479 bp S 5′-CCGGAAAAGAGGATGGCAATGAAT-3′ AS 5′CTGCTGAGCGCCCTGGAGAAC-3′ PECAM-1: 260 bp S 5′-GTCATGGCCATGGTCGAGTA-3′ AS 5′-CTCCTCGGCATCTTGCTGAA-3′ Flk-1: 239 bp S 5′-CACCTGGCACTCTCCACCTTC-3′ AS 5′-GATTTCATCCCACTACCGAAAG-3′

To examine the expression of CB1 and CB2 in mES cells, we performed RT-PCR analysis on control undifferentiated ES cells (Rosa26.6 and E14 ES cells) and on EBs derived from the secondary hematopoietic differentiation of these two ES cell lines at different time points as indicated. We found that CB1 and CB2 mRNAs and proteins were induced substantially in hematopoietic differentiated EBs as compared to control ES cells. As shown in Figure 1A and B, a significant induction of CB1 and CB2 gene expression was observed in day 8 and day 11 hematopoietic EBs from both Rosa26.6 and E14 ES cells, while undifferentiated mES cells had little expression of CB1 and CB2. Interestingly, expression of CXCR4 (a member of the GPCR family) was observed in undifferentiated ES cells and was not changed during ES cell differentiation (Fig. 1A). We also analyzed several hematopoietic markers in these hematopoietic EBs. We observed induction of Sca-1 expression, as well as induction of PECAM-1 and Flk-1 expression during ES cell differentiation (Fig. 1C), which is in agreement with other published reports [30].

**Figure 2:**
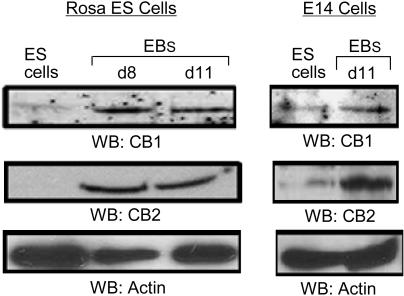
The expression of CB1 and CB2 receptors in Rosa26.6 and E14 ES cells as analyzed by Western blot analysis. Cells were lysed in RIPA buffer and 100 mg of total cell lysates were analyzed by SDS-PAGE, followed by Western blotting with CB1 or CB2 specific antibodies (at a dilution of 1:500). The cell lines 293T and SH-SY5Y were used as negative and positive controls, respectively, for CB1 expression. Actin was used as a control for loading. ES cells: undifferentiated ES cells; EBs: Embryoid bodies at different time points as indicated.

Next, CB1 and CB2 protein expression was analyzed in Rosa26.6 and E14 ES cells by Western blot analyses using two different specific sets of CB1 and CB2 antibodies, commercially available from Chemicon (set 1) (Fig. 2) and Sigma (set 2) (data not shown). Both sets of specific CB1 and CB2 antibodies showed induction of CB1 and CB2 protein expression during ES cell differentiation in day 8 and 11 EBs derived from secondary differentiation, as demonstrated by Western blot analysis (Fig. 2) and immunohistochemistry (data not shown). These results showed that CB1 and CB2 are both upregulated during the hematopoietic differentiation of ES cells and imply that CB1 and CB2 may have important regulatory roles in ES cell differentiation.

### Expression of endocannabinoids in mES cells and embryoid bodies derived from mES cells at days 7 and 14

**Figure 3:**
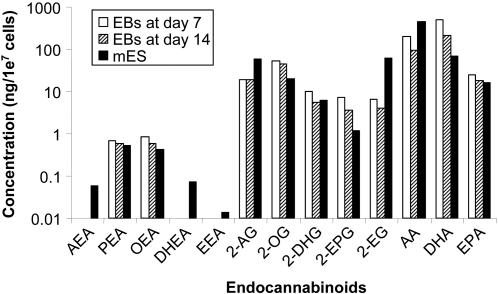
Comparison of endocannabinoid levels in mES cells and EBs at days 7 and 14, when the number of cells in each group is normalized to 10^7^ ( = 1e^7^). The groups depict the logarithms of each value. AEA, DHEA and EEA endocannabinoid levels were detected but were lower than the limit of quantitation (<0.05 ng/1e^7^ cells) for the number of cells analyzed.

To examine whether mES cells as well as EBs derived from mES cells express endocannabinoids, mES cells were analyzed for the expression of various fatty acids and their ethanolamide and monoglyceride derivatives using LC-APCI-MS analysis [31]. As shown in Figure 3, derivations of the endocannabinoids were detected and quantitated in mES cells and EBs at days 7 and 14. The level of anandamide (AEA) expression in the mES cells was much lower as compared to that of 2-AG, and AEA was not detected at all in EBs at days 7 and 14. The expression levels of: 2-AG, docosahexaenoic acid (DHA), arachidonic acid (AA), 2-oleoyl glycerol (2-OG), eicosapentaenoic acid (EPA), 2-docosahexaenoyl glycerol (2-DHG) and 2-eicosapentaenoyl glycerol (2-EPG), were abundant in mES cells, and EBs at days 7 and 14. Endocannabinoid levels in the embryonic stem cells were correlated to the number of mES cells (data not shown). These analyses showed that mES cells abundantly express endocannabinoids, specifically 2-AG which might be important for their survival. Furthermore, since both EBs at days 7 and 14 express endocannabinoids, this could suggest that endocannabinoids may play a role in the hematopoietic differentiation of mES cells.

### Effects of exogenous and endogenous cannabinoid ligands on the chemotaxis of mES cells

A major function of the 2-AG endocannabinoid is the stimulation of migration in B lymphocytes [32]. Since CXCR4 and its cognate ligand SDF-1α are involved in hematopoietic stem cell chemotaxis, migration and homing [33]–[45], and since CXCR4, CB1 and CB2 are members of the GPCR family, we therefore studied whether cannabinoid ligands act as chemotactic or chemokinetic agents for ES cells. We analyzed the effects of the endogenous cannabinoid ligand 2-AG, the exogenous ligand Δ^9^-THC and the specific CB2 receptor agonist, JWH-015, on the chemotaxis of undifferentiated ES cells as well as day 10 EBs derived from secondary hematopoietic differentiation.

**Figure 4:**
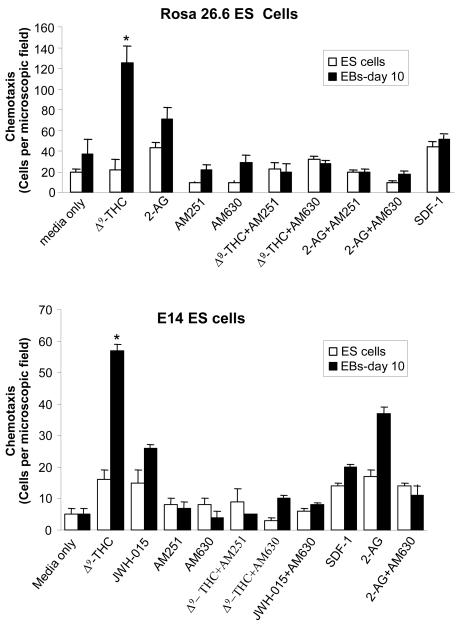
Effects of cannabinoid ligands on the chemotaxis of ES cells and hematopoietic differentiated ES-derived EB cells (EBs-day 10). Cells were placed in the upper well of the transwell in the presence or absence of specific inhibitors, as indicated. The ligands: 2-AG, Δ^9^-THC, JWH-015 and SDF-1α were placed in the lower chambers. Data show the mean value of 3 independent experiments (mean±SD). Error bars indicate SD. * P values with asterisk (*, P<0.05) show significant differences from control with media alone.

The chemotaxis assays were performed using Costar Transwells (Corning-Costar, Cambridge, MA). As shown in Figure 4, chemotaxis was observed with differentiated EBs at day 10 in the presence of the Δ^9^-THC, 2-AG and JWH-015 cannabinoid ligands, while the chemotaxis of undifferentiated ES cells was very low. This chemotaxis was inhibited by the CB1 and CB2 specific inhibitors, AM251 and AM630, respectively. Thus, cannabinoid ligands, such as 2-AG, exogenous Δ^9^-THC and JWH-015 induce the chemotaxis of hematopoietic differentiated ES-derived EB cells, mediated through both the CB1 and CB2 receptors.

### Effects of cannabinoid inhibitors on the survival of Rosa ES cells

**Figure 5:**
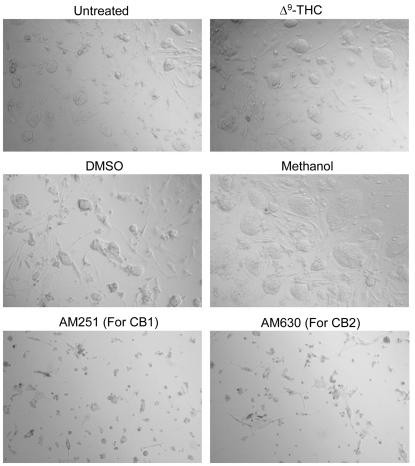
Effects of Δ^9^-THC and cannabinoid inhibitors (AM251 and AM630) on Rosa ES cell survival. Rosa ES cells were either untreated (as control) or treated with Δ^9^-THC, control DMSO (0.01%), control methanol (0.01%), or with the inhibitors AM251 (for the CB1 receptor) or AM630 (for the CB2 receptor), as indicated. After 48 hours, the cells were analyzed for their viability by light microscopy. This is a representative experiment out of three experiments.

To analyze the effects of Δ^9^-THC on the survival of Rosa ES cells, the Rosa ES cells were untreated or treated with Δ^9^-THC (1 µM) or with the specific inhibitors for CB1 (AM251) or CB2 (AM630) (in the absence of Δ^9^-THC) for 48 hours. In addition, Rosa ES cells were treated with DMSO (0.01%) or with methanol (0.01%) as vehicle controls. After 48 hours, cells were analyzed for viability. As seen in Figure 5, no effects on Rosa ES cell viability were observed upon treatment with DMSO or methanol as compared to the cannabinoid-treated ES cells. Δ^9^-THC also had no apoptotic effects on the Rosa ES cells. However, both inhibitors (AM251 and AM630) induced significant cell death in the absence of Δ^9^-THC (Fig. 5). These results suggest that endocannabinoids, either secreted by ES cells and/or by the Primary Embryonic Fibroblast (PEF) feeder cells, are important for the survival of ES cells and that specific inhibition of these endogenous ligands by inhibitors for CB1 and CB2 results in cell apoptosis.

### Effects of endocannabinoids and exogenous cannabinoid ligands on the differentiation of mES cells

**Figure 6:**
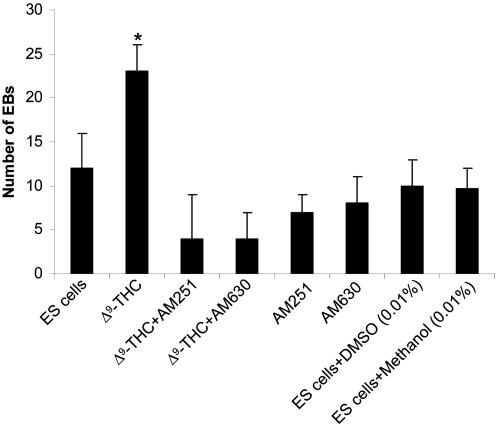
Effects of Δ^9^-THC on the differentiation of ES cells. Rosa ES cells were either untreated or treated with Δ^9^-THC in the presence or absence of cannabinoid inhibitors (AM630 and AM251), as indicated. After 14 days, the number of EBs was counted. Data represent the mean value of 3 independent experiments (mean±SD). * P values with asterisk (*, P<0.05) show significant differences from ES cells.

To examine the effects of exogenous cannabinoid ligands on ES cell differentiation, the ligand Δ^9^-THC (1 µM) was added to Rosa ES cells in DMEM medium. The CB1 specific inhibitor AM251 (1 µM) and the CB2 specific inhibitor AM630 (1 µM) were used for blocking the effects of cannabinoid ligands on ES cell differentiation, as indicated. The addition of AM251 or AM630 or addition of the control vehicle DMSO (0.01%) or methanol (0.01%) was performed during the primary differentiation stage and secondary hematopoietic differentiation of Rosa ES cells into EBs. ES cells were preincubated with AM251 or AM630 or with control vehicle DMSO or methanol for 30 min. The cells were then washed and further cultured for the *in vitro* hematopoietic differentiation over 14 days in the presence or absence of Δ^9^-THC, as described above. The number of EBs was counted after 14 days. As shown in Figure 6, Δ^9^-THC induced an increase in the number of EBs as compared to the control ES cells. However, when Δ^9^-THC was administered in the presence of AM251 or AM630, there was a decrease in the number of EBs (up to 70–75% inhibition). Interestingly, AM251 or AM630 alone also inhibited the number of EBs derived from ES cells (Fig. 6). This result suggests that these inhibitors block the effects on ES cell-derived EBs that are mediated by the endogenous endocannabinoid ligands, secreted by either the ES cells or PEF feeder cells, and that inhibition of CB1 and/or CB2 receptor-mediated effects, by specific CB1 and CB2 inhibitors, significantly blocks EB formation.

## Discussion

Recent work has linked changes in immune function to biologic and therapeutic targeting of cannabinoid receptors [13]. Cannabinoid receptor expression offers a new principle for regional immune homeostasis and disease susceptibility, and extends and refines the rationale for CB2-targeted immunotherapy in immune and inflammatory diseases. Therefore, elucidation of the effects of the cannabinoid system (especially CB2-transduced signaling) on stem cell self-renewal, proliferation, and differentiation should lead to the creation of new therapeutic approaches for hematological disorders as well as novel strategies involving pharmacological support for hematopoietic stem cell (HSC)-based therapies.

Here, we have characterized the expression and function of CB1 and CB2 cannabinoid receptors in murine ES cells and in ES-derived EBs, and examined the role of endocannabinoids and their cognate receptors, CB1 and CB2, as novel components of a new pathway important in murine ES cell differentiation. To test the hypothesis that the CB1 and CB2 receptors may have complementary roles in the hematopoietic differentiation of ES cells, we employed ES-derived differentiation methods using the Embryoid Body assay, which is well-controlled, easily manipulated and physiologically representative of the *in vivo* system. We demonstrated significant upregulation of CB1 and CB2 mRNA and protein in hematopoietic EBs at days 8 and 11 in both Rosa26.6 ES cells and E14 cells. The cannabinoid agonist Δ^9^-THC and the endocannabinoids induced the chemotaxis of EBs derived from either Rosa26.6 or E14 cells at day 10. Treatment of mES cells with the CB1 cannabinoid antagonist AM251 or with CB2 cannabinoid antagonist AM630 resulted in the death of these cells, indicating the involvement of endocannabinoids in mES cell survival. Murine ES cells were found to abundantly express endocannabinoids including the endocannabinoid 2-AG, which may play a role in mES cell survival. Furthermore, EBs at days 7 and 14 also express endocannabinoids, suggesting that endocannabinoids mediate the hematopoietic differentiation of mES cells, since the numbers of EBs derived from the mES cells was inhibited in the presence of AM251 and AM630. These results show that both CB1 and CB2 receptors, as well as their cognate agonists, are important regulators of mES cell survival and differentiation.

The availability of stem cells provides new approaches for the treatment of human diseases. Elucidation of the regulatory mechanisms responsible for stem cell differentiation is crucial for the application of ES cells to human diseases [46]. Mouse ES cells undergo unlimited self-renewal in the presence of the cytokine LIF, while retaining their multi-lineage differentiation capacity. Withdrawal of LIF and aggregation of cells lead to the differentiation of structures known as embryoid bodies (EBs). During differentiation, certain genes are upregulated and several others are downregulated in an intricately controlled fashion.

At each ES cell division, the alternative outcome of undergoing self-renewal or differentiation is decided by the interplay between intrinsic factors and extrinsic or selective signals. However, to date the intrinsic biology of these ES cells remains poorly defined. The stimulation of ES cell self-renewal was found to be restricted to LIF and related cytokines of the IL-6 family, which signal through the gp130 receptor via JAK kinase-mediated STAT3 activation [46]–[48]. PI3-kinase signaling was also observed to play an important role in mES cell survival and cell cycle progression [49]. Recently, STAT3 was reported to be the key downstream transcription factor of the LIF/gp130 signaling pathway in mES cells. Moreover, the Ca^2+^ signaling pathway in mES cells was also shown to mediate mES cell function [50]. Based on our results, we suggest that the cannabinoid system is an additional pathway involved in mES cell survival and differentiation.

The majority of directed differentiation protocols utilize an initial EB aggregation step. Therefore, the early-acting differentiation-promoting activities occurring inside the EBs are largely unknown. Based on our results, we suggest that exogenous cannabinoids can induce or promote hematopoietic differentiation. mES cells express both CB1 and CB2 receptors and both receptors are functional. Addition of exogenous selective cannabinoid agonists augmented the embryoid body formation derived from mES cells, indicating that cannabinoid ligands induced the hematopoietic differentiation of mES cells through CB1 and CB2 in both mES cells and EB-derived mES cells. Interestingly, CB2 receptors were recently found to promote mouse neural stem cell proliferation (NSCP) [47]. Cannabinoid agonists also increased *in vitro* NSCP proliferation and neurosphere generation [47]. The contribution of endocannabinoids to neurogenesis within the subventricular zone was recognized due to the reduced proliferation of neural precursors in CB1 receptor knockout mice [47]. Thus, these observations together with our results strongly suggest that both CB1 and CB2 activation are involved in the maintenance of mES cells and that the endocannabinoid system is essential in stem cell survival and stem cell hematopoietic differentiation.

## Materials and Methods

### Antibodies, and chemical and biological compounds

Anti-CB1 and anti-CB2 antibodies (ABR-Affinity BioReagents, Golden, CO) were used for immunostaining. The immunophenotyping of CB2 was confirmed with the use of another anti-CB2 antibody obtained from Sigma (St. Louis, MO). The cannabinoid ligands Δ^9^-THC (THC), JWH133, methanandamide, and CP55940 were also obtained from Sigma. ACEA and the cannabinoid receptor antagonists AM251 and AM630 were purchased from Tocris (Ellisville, MO). G-CSF (Neupogen) was obtained from Amgen Inc. (Thousand Oaks, CA). MethoCult 03434 (for mouse cells) was obtained from StemCell Technologies (Vancouver, BC, Canada). The deuterated endocannabinoids used as internal standards in the LC-APCI-MS analysis were synthesized in-house at the Center for Drug Discovery, Northeastern University (Boston, MA) following reported methods [31].

### RT-PCR analysis of CB1 and CB2 expression

RNA from total mES cells was extracted using the RNeasy Mini Kit (Qiagen, Valencia, CA) following the manufacturer's protocol. A QIAshredder spin column and DNase digestion were included in the isolation procedure to limit the possibility of PCR amplification of CB1 and CB2 from genomic DNA. cDNA and PCR amplification were performed with the BD Biosciences TITANIUM One-Step RT-PCR Kit using 200 ng of RNA as a template for first-strand synthesis. CB1 was amplified using primers: 5′-CGT GGG CAG CCT GTT CCT CA-3′ and 5′-CAT GCG GGC TTG GTC TGG-3′, which yield a product of 403 bp. CB2 was amplified using: 5′-CCG GAA AAG AGG ATG GCA ATG AAT-3′ and 5-CTG CTG AGC GCC CTG GAG AAC-3′, which yield a product of 479 bp. GAPDH was used as a positive control with primers: 5′-CTC ACT GGC ATG GCC TTC CG-3′ and 5′-ACC ACC CTG TTG CTG TAG CC-3′, which yield a product of 292 bp. The template was first denatured at 94°C for 2 min followed by 35 cycles (94°C for 30 sec, 58°C for 30 sec and 68°C for 1 min), followed by 68°C for 2 min in a myCycler Personal Thermal Cycler (Bio-Rad Laboratories, Inc). Aliquots (20 ml) of the PCR products were run on a 1.2% agarose gel containing 0.5 mg/ml ethidium bromide.

### Origination of embryoid bodies from ES cells

The Rosa26.6 ES cell line was obtained from Dr. Stuart Orkin (Children's Hospital, Harvard Medical School); The E14 and GFP-E14 cell lines were obtained from Dr. Bing Lim (Beth Israel Deaconess Medical Center, Boston). Culture and maintenance of ES cells in an undifferentiated state were performed as described previously [1]. Briefly, ES cells were maintained on a mouse PEF feeder cell line in ES medium containing Dulbecco's modified Eagle's medium (DMEM) with high glucose, 10 ng/ml murine leukemia inhibitory factor (mLIF; Chemicon International, Temecula, CA), 15% fetal calf serum (FCS; Hyclone, Logan, UT), 1 mM sodium pyruvate, 2 mM glutamine, 0.1 mM nonessential amino acid, 100 µM monothioglycerol (MTG; Sigma), 50 U/ml penicillin, and 10 µg/ml streptomycin. The ES cell lines were regularly analyzed, by using an ES cell characterization kit (Chemicon), for determination of alkaline phosphatase activity and detection of surface markers and transcription factors that are expressed by undifferentiated ES cells, such as Oct-4, Rex-1, SSEA-1 and Genesis (Fox D-3).

*In vitro* hematopoietic differentiation of ES cells was performed as described, essentially according to the protocol of StemCell Technologies. The embryoid body (EB) method involves two steps: first, spherical cell aggregates (termed embryoid bodies = EBs) are generated that contain ectodermal, mesodermal and endodermal derivatives ( = Primary Differentiation); second, these aggregates are selected for hematopoietic precursors and expanded with growth factors such as IL-3 and IL-6 ( = Secondary Hematopoietic Differentiation). Briefly, EBs were generated in 1% methylcellulose cultures (1×10^4^ ES cells per 35-mm Petri dish). To promote primary differentiation into EBs, ES cells were cultured in ES differentiation medium containing Iscove's modified Dulbecco's medium (IMDM), 15% FCS (StemCell Technologies), 2 mM glutamine, 150 µM MTG, and 40 ng/ml murine stem cell factor (mSCF). After 8 days of differentiation, the EBs were collected and washed. 1×10^4^ of single cells were seeded on 1% methylcellulose from the secondary hematopoietic differentiation medium. 15% FBS, 2 mM L-glutamate, 150 µM MTG, 20% BIT (10% BSA, 10 µg/ml insulin, 200 µg/ml transferrin), 150 ng/ml mSCF, 30 µg/ml IL-3, 30 µg/ml IL-6 and 3 U/ml Epo were added to the culture to promote hematopoietic differentiation. Cells were processed for Wright-Giemsa staining, RT-PCR and Western blot analyses at different times of EB culture differentiation, as indicated.

To determine the characteristics of various types of hematopoietic progenitors present during ES cell differentiation, EBs from ES cell lines were collected from the cultures at days 8 and 11 (from the day of replating) to obtain the hematopoietic progenitors. Cytospin preparation of these cells was stained with Wright-Giemsa and examined under a light microscope. Undifferentiated ES cells have a large nucleus, minimal cytoplasm, and one or more prominent dark nucleoli. Hematopoietic progenitors found in EB-day 14 cultures were identified by the morphology of erythroids, megakaryocytes, monocytes/macrophages, granulocytes and mast cells, as analyzed by field microscopy.

### Chemotaxis assays

The chemotaxis assays were performed using 5 µm-pore size and 6.5 mm-diameter Costar Transwells (Corning-Costar, Cambridge, MA), as previously described [30]. Cells were washed twice with Hank's balanced salt solution (HBSS) medium, resuspended in 100 µl medium [Iscove's Modified Dulbecco's Medium (IMDM) plus 0.5% BSA] and placed in the upper chamber of the Transwells. In the lower chamber, 600 µl of medium with or without ligand was placed, as indicated. After 4 hours of incubation at 37°C and 5% CO_2_, the upper chamber was removed and the number of migrated cells was determined using a CASY/TTC cell counter. The ligand Δ^9^-THC (Δ^9^-Tetrahydrocannabinol) and the endogenous ligand 2-AG (Cayman Chemical, Ann Arbor, MI, Catalog #62165) were added at 1 µM concentrations in IMDM media. The specific CB2 receptor agonist JWH-015 (Tocris Catalog number #1341) was also tested at a 1 µM concentration. The CB1 specific inhibitor AM251 (1 µM) (Tocris Catalog number #1117) and the CB2 specific inhibitor AM630 (1 µM) (Tocris Catalog number #1120) were used to block the effects of cannabinoid ligands on ES cell chemotaxis. For the inhibition studies, cells were preincubated with the inhibitor agonists for 30 min as indicated. SDF-1 alpha (25 ng/ml) was used as a positive control (PeproTech Inc., Catalog number #250-20A).

### Survival assays

2×10^4^ Rosa ES cells (per well of 96 wells), CB1 and CB2 specific ligands as well as inhibitors were added to the cell culture as indicated. A 1 µM final concentration was used for CP55940 (CB1 and CB2 agonists), ACEA (CB1 ligand) and JWH133 (CB2 ligand). A 1 µM final concentration of both AM251 (CB1 inhibitor) and AM630 (CB2 inhibitor) was used, as indicated. Cells were incubated for two days in a humidified CO_2_ atmosphere. The MTT assay was performed according to the Promega manual (Promega Cat# G5421), and the absorbance at 490 nm was then recorded.

### Endocannabinoid levels in embryonic stem cells

The extraction procedure for the calibration standards was performed as described [31]. Cells (mES cells, EBs at day 7 and EBs at day 14), at various concentrations as indicated, were homogenized in cold acetone:PBS, pH 7.4 (3∶1). The homogenates were sonicated for 30 seconds prior to centrifugation at 20,800 g for 5 minutes. The acetone from the resulting supernatants was removed under nitrogen. To the remaining supernatant, 50 µl PBS, one volume of methanol and two volumes of chloroform were added for liquid-liquid phase extraction of the lipids. The two phases were separated by centrifugation and the bottom organic layer was evaporated under nitrogen. The cell samples were reconstituted in 50 µl ethanol.

The system used for analysis was a TSQ Quantum Ultra triple quadrupole mass spectrometer (Thermo Electron, San Jose, CA) with an Agilent 1100 HPLC on the front end (Agilent Technologies, Wilmington, DE). The mobile phase consisted of 10 mM ammonium acetate (pH 7.3 using ammonium hydroxide; A) and 100% methanol (B). Separation of each analyte was achieved using a Zorbax SB-CN 2.1×50mm, 5 µm, 80Å, column (Agilent Technologies) and gradient elution; the autosampler was kept at 4°C to prevent analyte degradation [31]. Eluted peaks were ionized via atmospheric pressure chemical ionization (APCI) and detected by each analyte's SRM transition [31].

### Statistical analysis

The results are represented as the mean ± S.D. The significance of the data was determined by a two-tailed *t* test. *P*<0.05 was considered statistically significant.

## References

[1] Downing GJ, Battey JF (2004). Technical assessment of the first 20 years of research using mouse embryonic stem cell lines.. Stem Cells.

[2] Matsuoka S, Tsuji K, Hisakawa H, Xu Mj, Ebihara Y (2001). Generation of definitive hematopoietic stems cells from murine early yolk sac and paraaortic splanchnopleures by aorta-gonad-mesonephros region-derived stromal cells.. Blood.

[3] Metcalf D (2003). The unsolved enigmas of leukemia inhibitory factor.. Stem Cells.

[4] Viswanathan S, Benatar T, Rose-John S, Lauffenburger DA, Zandstra PW (2002). Ligand/receptor signaling threshold (LIST) model accounts for gp130-mediated embryonic stem cell self-renewal responses to LIF and HIL-6.. Stem Cells.

[5] Nishikawa SI, Nishikawa S, Hirashima M, Matsuyoshi N, Kodama H (1998). Progressive lineage analysis by cell sorting and culture identifies FLK1+VE-cadherin+ cells at a diverging point of endothelial and hemopoietic lineages.. Development.

[6] Choi K, Kennedy M, Kazarov A, Papadimitriou JC, Keller G (1998). A common precursor for hematopoietic and endothelial cells.. Development.

[7] Bautch VL, Stanford WL, Rapoport R, Russell S, Byrum RS (1996). Blood island formation in attached cultures of murine embryonic stem cells.. Dev Dyn.

[8] Vittet D, Prandini MH, Berthier R, Schweitzer A, Martin-Sisteron H (1996). Embryonic stem cells differentiate in vitro to endothelial cells through successive maturation steps.. Blood.

[9] Gudermann T, Nurnberg B, Schultz G (1995). Receptors and G proteins as primary components of transmembrane signal transduction. Part 1. G-protein-coupled receptors: structure and function.. J Mol Med.

[10] Savarese TM, Fraser CM (1992). In vitro mutagenesis and the search for structure-function relationships among G protein-coupled receptors.. Biochem J.

[11] Howard AD, McAllister G, Feighner SD, Liu Q, Nargund RP (2001). Orphan G-protein-coupled receptors and natural ligand discovery.. Trends Pharmacol Sci.

[12] Ferguson SS (2001). Evolving concepts in G protein-coupled receptor endocytosis: the role in receptor desensitization and signaling.. Pharmacol Rev.

[13] Piomelli D (2003). The molecular logic of endocannabinoid signalling.. Nat Rev Neurosci.

[14] Matsuda LA, Lolait SJ, Brownstein MJ, Young AC, Bonner TI (1990). Structure of a cannabinoid receptor and functional expression of the cloned cDNA.. Nature.

[15] Herkenham M, Lynn AB, Johnson MR, Melvin LS, de Costa BR (1991). Characterization and localization of cannabinoid receptors in rat brain: a quantitative in vitro autoradiographic study.. J Neurosci.

[16] Bouaboula M, Rinaldi M, Carayon P, Carillon C, Delpech B (1993). Cannabinoid-receptor expression in human leukocytes.. Eur J Biochem.

[17] Bouaboula M, Desnoyer N, Carayon P, Combes T, Casellas P (1999). Gi protein modulation induced by a selective inverse agonist for the peripheral cannabinoid receptor CB2: implication for intracellular signalization cross-regulation.. Mol Pharmacol.

[18] Galiegue S, Mary S, Marchand J, Dussossoy D, Carriere D (1995). Expression of central and peripheral cannabinoid receptors in human immune tissues and leukocyte subpopulations.. Eur J Biochem.

[19] Daaka Y, Klein TW, Friedman H (1995). Expression of cannabinoid receptor mRNA in murine and human leukocytes.. Adv Exp Med Biol.

[20] Schatz AR, Lee M, Condie RB, Pulaski JT, Kaminski NE (1997). Cannabinoid receptors CB1 and CB2: a characterization of expression and adenylate cyclase modulation within the immune system.. Toxicol Appl Pharmacol.

[21] Guzman M (2003). Cannabinoids: potential anticancer agents.. Nat Rev Cancer.

[22] Buckley NE, McCoy KL, Mezey E, Bonner T, Zimmer A (2000). Immunomodulation by cannabinoids is absent in mice deficient for the cannabinoid CB(2) receptor.. Eur J Pharmacol.

[23] Abel E (1980). Marijuana: the first twelve thousand years..

[24] Alger BE (2004). Endocannabinoids: getting the message across.. Proc Natl Acad Sci U S A,.

[25] Pertwee RG (1999). Pharmacology of cannabinoid receptor ligands.. Curr Med Chem.

[26] Huffman JW, Liddle J, Yu S, Aung MM, Abood ME (1999). 3-(1′,1′-Dimethylbutyl)-1-deoxy-delta8-THC and related compounds: synthesis of selective ligands for the CB2 receptor.. Bioorg Med Chem.

[27] Hillard CJ, Manna S, Greenberg MJ, DiCamelli R, Ross RA (1999). Synthesis and characterization of potent and selective agonists of the neuronal cannabinoid receptor (CB1).. J Pharmacol Exp Ther.

[28] Abadji V, Lin S, Taha G, Griffin G, Stevenson LA (1994). (R)-anandamide: a chiral novel anandamide possessing higher potency and metabolic stability.. J Med Chem.

[29] Chin CN, Murphy JW, Huffman JW, Kendall DA (1999). The third transmembrane helix of the cannabinoid receptor plays a role in the selectivity of aminoalkylindoles for CB2, peripheral cannabinoid receptor.. J Pharmacol Exp Ther.

[30] Jorda MA, Verbakel SE, Valk PJ, Vankan-Berkhoudt YV, Maccarrone M (2002). Hematopoietic cells expressing the peripheral cannabinoid receptor migrate in response to the endocannabinoid 2-arachidonoylglycerol.. Blood.

[31] Williams J, Wood J, Makriyannis A (2007). A quantitative method for the profiling of the endocannabinoid metabolome by LC-APCI-MS.. Analytical Chemistry.

[32] Quesenberry PJ, Colvin G, Abedi M (2005). Perspective: fundamental and clinical concepts on stem cell homing and engraftment: a journey to niches and beyond.. Exp Hematol.

[33] Mahmud N, Patel H, Hoffman R (2004). Growth factors mobilize CXCR4 low/negative primitive hematopoietic stem/progenitor cells from the bone marrow of nonhuman primates.. Biol Blood Marrow Transplant.

[34] Hart C, Drewel D, Mueller G, Grassinger J, Zaiss M (2004). Expression and function of homing-essential molecules and enhanced in vivo homing ability of human peripheral blood-derived hematopoietic progenitor cells after stimulation with stem cell factor.. Stem Cells.

[35] Nilsson SK, Simmons PJ (2004). Transplantable stem cells: home to specific niches.. Curr Opin Hematol.

[36] Wynn RF, Hart CA, Corradi-Perini C, O'Neill L, Evans CA (2004). A small proportion of mesenchymal stem cells strongly expresses functionally active CXCR4 receptor capable of promoting migration to bone marrow.. Blood.

[37] Kimura T, Boehmler AM, Seitz G, Kuci S, Wiesner T (2004). The sphingosine 1-phosphate receptor agonist FTY720 supports CXCR4-dependent migration and bone marrow homing of human CD34+ progenitor cells.. Blood.

[38] Juarez J, Endall L (2004). SDF-1 and CXCR4 in normal and malignant hematopoiesis.. Histol Histopathol.

[39] Adams GB, Chabner KT, Foxall RB, Weibrecht KW, Rodrigues NP (2003). Heterologous cells cooperate to augment stem cell migration, homing, and engraftment.. Blood.

[40] Kollet O, Petit I, Kahn J, Samira S, Dar A (2002). Human CD34(+)CXCR4(-) sorted cells harbor intracellular CXCR4, which can be functionally expressed and provide NOD/SCID repopulation.. Blood.

[41] Plett PA, Frankovitz SM, Wolber FM, Abonour R, Orschell-Traycoff CM (2002). Treatment of circulating CD34(+) cells with SDF-1alpha or anti-CXCR4 antibody enhances migration and NOD/SCID repopulating potential.. Exp Hematol.

[42] Peled A, Hardan I, Trakhtenbrot L, Gur E, Magid M (2002). Immature leukemic CD34+CXCR4+ cells from CML patients have lower integrin-dependent migration and adhesion in response to the chemokine SDF-1.. Stem Cells.

[43] Voermans C, van Hennik PB, van der Schoot CE (2001). Homing of human hematopoietic stem and progenitor cells: new insights, new challenges?. J Hematother Stem Cell Res.

[44] Shen W, Bendall LJ, Gottlieb DJ, Bradstock KF (2001). The chemokine receptor CXCR4 enhances integrin-mediated in vitro adhesion and facilitates engraftment of leukemic precursor-B cells in the bone marrow.. Exp Hematol.

[45] Peled A, Petit I, Kollet O, Magid M, Ponomaryov T (1999). Dependence of human stem cell engraftment and repopulation of NOD/SCID mice on CXCR4.. Science.

[46] Rao M (2004). Conserved and divergent paths that regulate self-renewal in mouse and human embryonic stem cells.. Dev Bio.

[47] Molina-Holgado F, Rubio-Araiz A, Garcia-Ovejero D, Williams RJ, Moore JD (2007). CB2 cannabinoid receptors promote mouse neural stem cell proliferation.. Eur J Neurosci.

[48] Thorsten G, Timothy EA (2003). Pharmacological potential of embryonic stem cells.. Pharmacol Res.

[49] Burdon T, Smith A, Savatier P (2002). Signalling, cell cycle and pluripotency in embryonic stem cells.. Trends Cell Biol.

[50] Yanagida E, Shoji S, Hirayama Y, Yoshikawa F, Otsu K (2004). Functional expression of Ca2+ signaling pathways in mouse embryonic stem cells.. Cell Calcium.

